# Mental health and quality of life in different obesity phenotypes: a systematic review

**DOI:** 10.1186/s12955-022-01974-2

**Published:** 2022-04-19

**Authors:** Behnaz Abiri, Farhad Hosseinpanah, Seyedshahab Banihashem, Seyed Ataollah Madinehzad, Majid Valizadeh

**Affiliations:** 1 Obesity Research Center, Research Institute for Endocrine Sciences, Shahid Beheshti University of Medical Sciences, Tehran, Iran; 2 Taleghani Hospital Research Development Committee (Taleghani-HRDC), Shahid Beheshti University of Medical Sciences, Tehran, Iran

**Keywords:** Obesity phenotype, Mental health, Health-related quality of life, Metabolic phenotype

## Abstract

**Objectives:**

It has been suggested that obesity phenotypes are related to mental health problems and health-related quality of life (HRQoL). However, there is no certain consensus. This systematic review aimed to evaluate the association between different obesity phenotypes with common psychiatric symptoms and HRQoL.

**Methods:**

Electronic databases i.e. PubMed, Scopus, EMBASE, and google scholar were searched until September 2021, to identify studies that investigated associations between the obesity phenotypes with psychiatric symptoms and/or mental and physical HRQoL. Two researchers independently checked titles and abstracts, evaluated full-text studies, extracted data, and appraised their quality using the Newcastle–Ottawa Scale.

**Results:**

Eighteen studies, with a total of 3,929,203 participants, were included. Of the studies included in this systematic review, 10 articles evaluated the association between obesity phenotypes and psychiatric symptoms, while six papers investigated the association between HRQoL and obesity phenotypes, and two studies assessed both. As a whole, the findings of these studies suggest that obese individuals with a favorable metabolic profile have a slightly higher risk of mental health problems and poor quality of life, however, the risk becomes larger when obesity is combined with an adverse metabolic profile. So, metabolically healthy obesity may not be a completely benign condition in relation to mental disorders and poor quality of life.

**Conclusion:**

According to published research, obesity is likely to increase the risk of mental health problems and poor quality of life when metabolic disturbances are present.

## Background

Obesity is a worldwide problem characterized by excess body fat accumulation; the incidence is on the rise [1]. Worldwide, the prevalence of overweight and obesity has doubled since 1980, and a third of the population is obese or overweight all over the world now [2]. Obesity is connected with cardiometabolic diseases, such as hypertension, diabetes mellitus, dyslipidemia, and cardiovascular diseases (CVDs) [1]. Additionally, the obesity-related insulin resistance and metabolic disturbances can have adverse effects on the cardiometabolic system, which may in turn influence mental health and health-related quality of life (HRQoL) [1].

Individuals with obesity do not always have metabolic abnormalities, and individuals in the normal weight range do not always have favourable metabolic responses [3]. Hence, it has been suggested that obesity phenotypes are classified based on metabolic state such as metabolically healthy but obese (MHO), metabolically abnormal but of normal weight (MANW), and metabolically unhealthy and obese (MUHO) [3].

The relationship between obesity phenotypes and quality of life (QoL) and mental health has been examined in some literature [4–7]; but, QoL and mental health issues associated with obesity phenotypes have not been studied as thoroughly as physical difficulties [8]. Despite a previous meta-analysis of prospective studies suggesting that individuals with higher body mass index (BMI) have a greater chance of developing depression [9], some studies find no relationship between obesity and depression [10], and one study reported lower mental health risks associated with higher BMI [11]. The metabolic syndrome (MetS), on the other hand, leads to health conditions that are unfavourable; therefore, people with MetS tend to have lower overall health-related quality of life [12]. There is a discrepancy amongst findings of the unfavorable relationship between HRQoL and MetS, with some reporting a negative association between women [13–15], men [16], or reporting even better HRQoL amongst those with MetS [17] or no relationship at all [18, 19].

Yet, the impact of obesity phenotypes on QoL and mental health outcomes, including stress, anxiety, and depression, remains unclear. These conditions influence individuals’ moods or feelings, reduce productivity, and lead to an enormous economic burden [8].

It would be helpful to understand how metabolic phenotypes relate to mental health and HRQoL for individuals with MHO as well as those who present as MANW in terms of health promotion and policies. So, in this systematic review, we examine the relationship between obesity phenotypes with mental health and HRQoL.

## Methods

### Search strategy

**Fig. 1:**
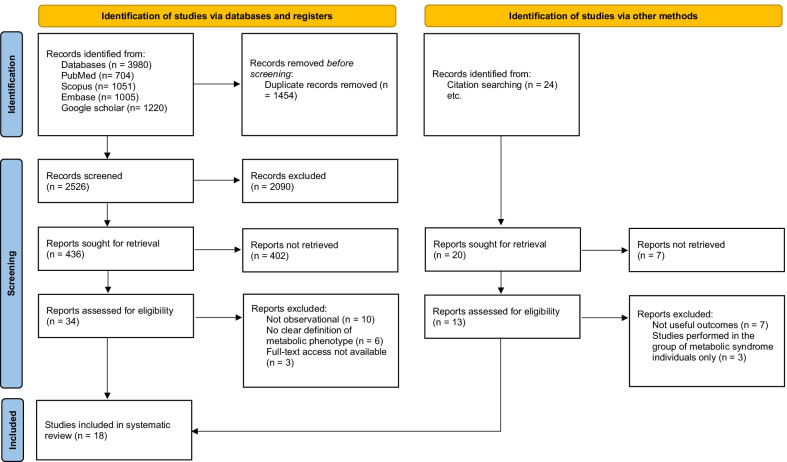
PRISMA flow diagram for selection process of the studies

The systematic review question was “what is the relationship between obesity phenotypes with common psychiatric symptoms and HRQoL?” A literature review was done in PubMed, Scopus, EMBASE, and google scholar databases until September 2021, with no restrictions on language and date. The following search terms were used in this search: metabolically AND (healthy OR unhealthy OR benign) AND (overweight OR obes* OR “over weight”) AND phenotype AND (depression OR depress* OR “depressive disorder” OR mood OR stress OR emotion OR anxiety OR mental health) AND (quality of life OR health-related quality of life). We searched keywords in PubMed using both [tiab] and [MeSH] tags. The reference lists of the retrieved papers were also scanned to ensure no data had been missed. To find relevant studies missed by the electronic search strategy, citation tracing for included studies was also performed. The citation tracing process lasted until September 2021. All potentially eligible studies were included in the review, regardless of primary outcome or language. All included articles were published in English. The selection process is presented in Fig. 1. Because of the diversity in the comparisons of the included studies (differences in outcomes, exposures, participants, and settings) and lack of data amenable to analysis and pooled size, we conducted a qualitative systematic review. The systematic review was carried out following the Preferred Reporting Items for Systematic Reviews and Meta-Analyses (PRISMA 2020) Statement [20].

### Eligibility criteria

Observational studies (cohort, cross-sectional, and case–control studies) were considered, and on the other hand, clinical trials, reviews, editorials, and studies on non-human models as well as studies without full text access were excluded. We did not specify a strict age range since the number of eligible studies was limited. The studies involved evaluating the association between different obesity phenotypes with mental health outcomes and QoL.

### Study selection

Following the elimination of duplicates, titles and abstracts collected in the initial search were evaluated separately by two authors (BA and FH). Full-text articles were assessed by these two authors to be assured they matched the eligible inclusion and exclusion criteria. Researchers were consulted about any disagreements they had.

### Data extraction and quality assessment

A data mining sheet was created to record information about: first author, year of publication, study design, number and characteristics of participants, exposure assessment, outcomes, and main findings. Details of data extraction and critical appraisal of the studies are demonstrated in Table 1. The Newcastle–Ottawa Scale (NOS) for evaluating quality of observational studies was used [21]. The scale contained eight items ranging from zero to nine, pertaining to evaluation of selection, comparability, and outcome or exposure. The quality assessment scores of articles are shown in Table 2.

**Table 1:** Characteristics of the studies on the association between obesity phenotypes with mental health and quality of life

First author (Reference No)	Year of publication	Study design	No of participants (sex)	Age of participants	Exposure assessment	Outcome assessment	Main finding
Mehrabi [8]	2021	Cross-sectional	2469 (male and female)	46.2 ± 15.9	Obesity was defined as BMI ≥ 30 kg/m^2^, and MUH status based on having MetS or T2DM	Emotional states were assessed by the Persian version of DASS-21	Men and women with various obesity phenotypes experienced different anxiety and stress levels. While MUHO women and all MUH men experienced more anxiety and stress levels than MHNO individuals, none of the obesity phenotypes were associated with depression
Portugal-Nunes [24]	2021	Cross-sectional	101 (male and female)	64 ± 8.46	The anthropometric measures included weight (Kg), height (m), and abdominal perimeter (cm). FBG, fasting insulin, TG, and HDL were measured	Mood was assessed by the Geriatric Depression Scale (GDS, long-version)	The association of metabolic dysfunction with depressive mood is influenced by age
Park and Lee [29]	2021	Cross-sectional	288,044 (male and female)	≥ 18 years	The MUH group was defined as those who have one of the following characteristics: FBG > 100 mg/dL or current use of hypoglycemic medication, BP ≥ 130/85 mmHg or current use of BP medication, TG ≥ 150 mg/dL or the use of antilipidemic medication, low HDL-C (< 40 mg/dL for men and < 50 mg/dL for women), and HOMA-IR score ≥ 2.5. MH was defined as those who do not meet the above criteria	Depression was assessed by the CES-D scale	The metabolic phenotype exerts a direct influence on emotional problems. Metabolic health may be used as an indicator of mental health
Kim [1]	2020	Cross-sectional	6057 (male and female)	≥ 20 years	Normal weight or obese was assessed by BMI. MUH status was defined as the presence of any three or more of the revised NCEP-ATP III definitions of MetS	Psychiatric symptoms including sleep time, stress, depression, suicide thoughts, were assessed by asking the related questions. Health related quality of life was evaluated by the EQ-5D	With or without metabolic abnormalities, obesity is associated with mental health problems and decreased quality of life
Seo [25]	2020	Longitudinal	3,586,492 adult individuals (male and female)	40–70 years	Obesity was defined as BMI ≥ 25 kg/m^2^ and MH as MetS risk < 2	Depression was determined by a recording of ICD-10 codes F32.0 to F34.9 on health insurance data or the taking of antidepressant	MUHO has a higher risk of depressive symptoms than MHN. Furthermore, in women participants, MHO is also related to a higher risk of depressive symptoms. MHO is not a totally benign condition in relation to depression in women
Imbiriba [26]	2020	Cross-sectional	2371 (male and female)	49.6 ± 7.1 years	Metabolic profile classification was based on the Third NHANES criteria for anthropometric–metabolic profiles	Mental health data were collected through the Portuguese version of the CIS-R	There was a significant association between low skill discretion and an adverse metabolic profile in models adjusted for age, sex and race. No associations were significant between job stress domains and the metabolic profile of obese individuals in full models
Delgado [23]	2018	Cross-sectional	125 (100 obese, 25 non-obese) (male and female)	Obese subjects: 39.5 (10.5) years Non-obese subjects: 39.9 (10.4) years	MUO was defined as obesity associated with two or more metabolic alterations, including low HDL, hypertriglyceridemia, high FBG and hypertension	Depression was assessed using the Montgomery-Asberg Depression Rating Scale (MADRS) and Mini-International Neuropsychiatric Interview (MINI)	Inclusion of inflammation in the definition of MUO drives the association found between poor metabolic health and depressive symptoms
Amiri [32]	2018	Cross-sectional	2880 (male and female)	> 19 years	Weight status was assessed by BMI. Based on the JIS definition, metabolic syndrome is defined as the presence of any 3 of the following five risk factors: (1) abdominal obesity; (2) reduced HDL-C < 50 mg/dl in women, < 40 in men or on drug treatment; (3) high TG levels ≥ 150 mg/dl or on drug treatment; (4) high BP or drug treatment; (5) high FBG ≥ 100 mg/dl or on drug treatment	HRQoL was assessed using the Short-Form 12-Item Health Survey version 2 (SF-12v2)	Compared to those with normal weight normal metabolic status, only obese dysmetabolic individuals were more likely to report poor physical HRQoL in both genders
Yosaee [27]	2018	Cross-sectional	157 adult subjects (male and female)	20–55 years	MUHO, MHO and non-obese metabolically healthy, diagnosed according to the NCEP-ATP III criteria and BMI	Depressive symptoms assessed by BDI	MHO was a benign phenotype in relation to depression
Truthmann [22]	2017	Cross-sectional	3298 subjects (male and female)	18–79 years	MHNO, MUNO, MHO, and MUO were defined by ATPIII criteria and BMI	Physical HRQoL was measured by the Short Form-36 version 2 PCS score	Obesity was significantly related to lower physical HRQoL, independent of metabolic health status, especially among women
Hinnouho [30]	2017	Longitudinal	14,475 subjects (male and female)	44–59 years	Obesity was defined as BMI ≥ 30 kg/m2 and metabolic health as having none of the self-reported following CV risk factors: hypertension, T2DM and dyslipidemia	Depressive symptoms were assessed by the Center For CES-D scale	Poor metabolic health, irrespective of BMI was associated with more depression at the baseline, whereas a poorer course of depression over time was observed only in those with both obesity and poor metabolic health
Lopez-Garcia [5]	2017	Longitudinal	4397 individuals (male and female)	≥ 18 years	Weight was assessed by BMI. Two metabolic statuses 9 were defined: healthy (0–1 CA) and unhealthy (≥ 2 CA)	HRQoL was measured with the PCS and the MCS of the SF-12 questionnaire	Both obesity and CA should be addressed to improve HRQoL
Donini [4]	2016	Cross-sectional	253 subjects (male and female)	18–65 years	MHO and MUO were defined based on the absence or the presence of the MetS, respectively. PA was assessed by IPAQ questionnaire	HRQoL was measured with the SF-12 questionnaire	The metabolic comorbidity and the impairment of functional ability and psycho-social functioning may have a different timing in the natural history of obesity
Yang [12]	2016	Cross-sectional	6217 men and 8243 women	Over 30 years	Metabolic abnormality was defined by the criteria of the NCEP-ATP III	HRQoL was evaluated using the EQ-5D questionnaire	The MANW is the least favorable state of HRQoL for men. In women, the MUHO and MHO groups had the most adversely affected HRQoL
Phillips and Perry [7]	2015	Cross-sectional	2047 middle-aged male and female	50–69 years	MH was defined by three definitions based on a range of CA including MetS criteria, insulin resistance and inflammation	Depression, anxiety and well-being were assessed using the CES-D, the HADS and the WHO-5 Well Being Index	A favourable metabolic profile is positively related to mental health in obese middle-aged adults, but findings were dependent on MH definition
Hamer [28]	2012	Longitudinal	3851 subjects (male and female)	63.0 ± 8.9 years	Based on BP, HDL, TG, glycated haemoglobin, and CRP, subjects were classified as ‘MH’ (0 or 1 metabolic abnormality) or ‘MU’ (≥ 2 metabolic abnormalities)	Depressive symptoms were assessed using the 8-item CES-D scale	The association between obesity and risk of depressive symptoms seems to be partly dependent on metabolic health
Ul-Haq [31]	2012	Cross-sectional	5608 subjects (male and female)	≥ 20 years	Metabolic comorbidity was defined as the presence of one or more of these conditions: diabetes, HTN, hypercholesterolemia or CVD	HRQoL was evaluated using the Scottish Health Survey	The adverse impact of obesity on HRQoL is greater among individuals with metabolic comorbidity
Tsai [6]	2008	Cross-sectional	361 overweight and obese subjects (male and female)	No MetS: 44.9 ± 10.0 MetS: 48.2 ± 9.5	The presence of MetS was assessed using the NCEP criteria	HRQoL was measured with the SF-36 questionnaire. Depression was assessed using the BDI	Individuals with MetS reported lower HRQoL. This appeared to be an effect of increased weight, rather than a unique effect of MetS

BMI, body mass index; T2DM, type 2 diabetes mellitus; MetS, metabolic syndrome; MUH, metabolically unhealthy; MUHO, metabolically unhealthy obesity; DASS-21, depression, anxiety, and stress scale-21; MHNO, metabolically healthy non-obese; FBG, fasting blood glucose; TG, triglycerides; HDL, high-density lipoprotein; BP, blood pressure; MH, metabolically healthy; CES-D, Center for Epidemiologic studies depression; NCEP-ATP III, national cholesterol education program adult treatment panel; EQ-5D, EuroQol five-dimension; ICD, international classification of disease; NHANES, national health and nutrition examination survey; CIS-R, Clinical Interview Schedule-Revised; MUNO, metabolically unhealthy non-obese; HRQol, health related quality of life; BDI, beck depression inventory; PCS, physical component summary; CV, cardiovascular; CA, cardiometabolic abnormality; MCS, mental component summary; PA, physical activity; IPAQ, international physical activity questionnaire; HADS, hospital anxiety and depression scale; WHO, world health organization; MANW, metabolically abnormal but normal weight; CRP, C-reactive protein; MU, metabolically unhealthy; CVD, cardiovascular disease; HTN, hypertension; JIS, Joint Interim Statement

**Table 2:** Quality evaluation of the included studies

References	Selection	Comparability	Outcome	Total
Mehrabi et al. [8]	**	**	**	******
Portugal-Nunes et al. [24]	**	*	**	*****
Park and Lee et al. [29]	***	**	**	*******
Kim et al. [1]	**	**	**	******
Seo et al. [25]	***	***	**	********
Imbiriba et al. [26]	**	**	*	*****
Delgado et al. [23]	**	*	*	****
Amiri et al. [32]	**	**	*	*****
Yosaee et al. [27]	*	**	*	****
Truthmann et al. [22]	**	**	*	*****
Hinnouho et al. [30]	**	***	***	********
Lopez-Garcia et al. [5]	**	***	**	*******
Donini et al. [4]	*	**	**	*****
Yang et al. [12]	*	**	**	*****
Phillips et al. [7]	****	**	**	********
Hamer et al. [28]	**	**	**	******
Ul-Haq et al. [31]	**	**	**	******
Tsai et al. [6]	*	*	**	****

## Results

### Study characteristics

Initially, 3980 studies were found from databases. Among which, after removing 1454 duplicate articles, 2073 were excluded after scanning the titles/abstracts because they did not relate to the present systematic review. After carefully screening of 453 full texts, we also excluded 435 more studies because they investigated the association between metabolic phenotype with an outcome other than mental health or HRQoL, or were animal or in vitro studies in design, editorial, and reviews. Finally, 18 different studies [1, 4–8, 12, 22–32] with a total of 3,929,203 participants, published between 2008 and 2021, were eligible for the systematic review. The flow chart of study selection is presented in Fig. 1.

The sample size of the included studies ranged between 101 and 3,586,492 subjects. The age of participants was ≥ 18 years old.

Among the included articles, all of the studies involved both sexes. Four studies were longitudinal [5, 25, 28, 30] and others were cross-sectional [1, 4, 6, 8, 12, 22–24, 26, 27, 29–32]. Table 1 summarizes the characteristics of all included studies.

### Outcome assessment

Data obtained included 18 studies with 10 articles on mental health dimensions [7, 8, 23–30], six papers with data on health-related quality of life [4, 5, 12, 22, 31, 32] and two studies investigated both mental health and QoL in different metabolic phenotypes [1, 6]. Depression is the outcome in 8 studies [6, 7, 23, 25, 27–30] and was assessed by different tools: the Center for Epidemiologic Studies Depression scale (CES-D) in four studies [7, 28–30], Beck Depression Inventory (BDI) in two studies [6, 27], Geriatric Depression Scale (GDS) in one study [24], Montgomery-Asberg Depression Rating Scale (MADRS) and Mini-International Neuropsychiatric Interview (MINI) in another study [23], and International Classification of Disease (ICD-10) in one study [25]. In one paper [7] anxiety and well being were assessed by Hospital Anxiety and Depression Scale (HADS) and World Health Organization (WHO) well being index, respectively. In one study [8] emotion state was assessed using Depression, Anxiety, and Stress Scale-21 (DASS-21). Psychiatric symptoms were assessed by asking the related questions, in one study [1]. Quality of life is the outcome in eight studies [1, 4–6, 12, 22, 31, 32] and was assessed by different scales: the Short Form (SF-36 and SF-12) in six studies [4–6, 8, 22, 32], EuroQol-5 dimension questionnaire (EQ-5D) in two papers [1, 12], and Scottish health survey in one study [31].

### The association between obesity phenotypes with mental health and HRQoL

In a study by Mehrabi et al. [8], between 2469 men and women it was demonstrated that after adjustment for probable confounders, compared to MHNW men, in metabolically unhealthy men, anxiety levels are significantly higher regardless of whether they are obese (OR 1.78, 95% CI 1.25–2.54; *P* ≤ 0.001) or not (OR 1.61, 95% CI 1.17–2.21; *P* ≤ 0.001), and also in MUHO women (OR 1.73, 95% CI 1.28–2.34; *P* ≤ 0.001) compared to MHNW women. Additionally, Men who are MUNOs are significantly more likely to experience stress than those who are MHNWs (OR 1.40, 95% CI 1.02–1.90; *P* = 0.04), and women who are MUHO have significantly higher stress levels than those who are MHNW (OR 1.45, 95% CI 1.07–1.96; *P* = 0.02). Researchers found that mean anxiety scores in men and mean anxiety and stress scores in women were significantly different among obese phenotypes (*P* = 0.044, *P* = 0.02, and *P* = 0.022, respectively). After adjustment for probable confounders, such as age, marital state, education, job state, smoking state, and physical activity, the odds of having higher levels of anxiety were considerably greater in MUHO (OR 1.78, 95% CI 1.25, 2.54; *P* ≤ 0.001) and MUNW men (OR 1.61, 95% CI 1.17, 2.21; *P* ≤ 0.001) compared to MHNW men, and also in MUHO women (OR 1.73, 95% CI 1.28, 2.34; *P* ≤ 0.001) compared to MHNW women. Moreover, MUNW men (OR 1.40, 95% CI 1.02, 1.90; *P* = 0.04) and MUHO women (OR 1.45, 95% CI 1.07, 1.96; *P* = 0.02) were significantly more likely to have higher stress levels compared to MHNW men and women, respectively. In MUHO women, higher depression levels were observed before adjustment (OR 1.39, 95% CI 1.04, 1.84; *P* = 0.02), but there was no difference after adjustment in either gender.

In another study [23], stratified by metabolic state (MHO or MUHO), there was no substantial difference in MADRS scores reported for obese patients. (t = 0.8, *P* = 0.4). Aditionally, Depression and non-depression were not associated with significant differences in MUHO prevalence or metabolic disturbances.

Another cross-sectional analysis [24] concluded that an abnormal glucose or lipid metabolism was linearly related to depressive symptoms, and excess weight was U-shaped in its relationship to depression. Glucose dysmetabolism, obesity, and metabolic disturbances are positively related to depression among the younger subjects in our sample and disappear with age. In the respective models, metabolic abnormalities (β = 0.066, *P* = 0.029), disturbances in glucose (β = 0.062, *P* = 0.039), and lipids metanolism (β = 0.076, *P* = 0.011) were significantly related to a greater score in GDS.

An investigation on a sample of 3,586,492 adults from the National Health Insurance Database of Korea [25], was reported that MUHO subjects, regardless of gender, suffered the most from incident depression (OR = 1.01 in male; OR = 1.09 in female). Also, in women, depression risk is higher among those with MHO. It was found that only MUHO subjects were at a higher risk of experiencing depression (OR = 1.012; CI = 1.002, 1.023). In compared to all subjects without obesity, the risk of depressive mood for MUHO (OR = 1.096; CI = 1.085, 1.107) was greater than for MHO individuals (OR = 1.073; CI = 1.061, 1.086).

In a study [26] on 2371 obese individuals at the Brazilian Longitudinal Study of Adult Health (ELSA-Brasil), the findings after adjusting for race, age, and gender showed low skill discretion to be related to MUHO. But, in fully-adjusted models, the MUHO phenotype was not related to high job demand (OR = 1.05; 95% CI 0.82–1.35), low skill discretion (OR = 1.26; 95% CI 0.95–1.68), low decision power (OR = 0.94; 95% CI 0.70–1.25) nor low social support (OR = 0.93; 95% CI 0.71–1.20).

A comparison was made by Yosaee et al. [27] between depressed MUHO and a healthy control group. The metabolically healthy obese and nonobese group had significantly lower BDI scores (*P* = 0.036) than the MUHO group after adjusting for gender, marital status, and educational level.

Another research [28] revealed that MUHO subjects had a greater risk of depression in follow-up (OR = 1.50, 95% CI, 1.05–2.15) than non-obese healthy subjects after adjustment for baseline CES-D scores and other variables, though the MHO did not. (OR = 1.38, 95% CI, 0.88–2.17).

Park et al. [29] reported that even after controlling for sleep, depression, and metabolic parameters, women older than 40 years had a low suicide risk in the metabolically healthy group (OR = 0.812, 95% CI = 0.663–0.993). Suicide risk was considerably higher among women over 40 years old in the metabolically unhealthy group when other covariates weren't adjusted (OR = 1.535, 95% CI = 1.180–1.998). Among men of all ages, there did not seem to be a significant difference in risk factors for suicide depending on metabolic health or unhealthy metabolism.

MUHO participants were more likely than MHNO participants to suffer from anxiety and depression, according to another cross-sectional study conducted on 2047 middle-aged Irish men and women. [7], demonstrated that compared to the MHNO participants the risk of anxiety and depression was higher in the MUHO group (OR 1.63–1.66, OR 1.82–1.83 for anxiety and depressive mood, respectively, according to the definition of metabolic health). MHO subjects did not appear to be at greater risk for these conditions.

Hinnouho et al. [30] in their investigation on a sample of 14,475 men and women, in the Gazel cohort, revealed that metabolically unhealthy normal weight [OR 1.37; 95% CI 1.25 ± 1.51], overweight [1.44 (1.31 ± 1.59)] and obese [1.30 (1.10 ± 1.54)] but not MHO subjects [1.04 (0.81 ± 1.32)] had more depression risk at the beginning of follow-up compared to MHNW groups.

In MHNW individuals, individual's levels of depression declined over time [0.52 (0.50 ± 0.55)], whereas MUHO respondents were less affected [1.22 (1.07 ± 1.40)]. Participants in the MUHO study had a higher risk of depressive symptoms at the beginning of follow-up compared to those participating in MHO, but this risk declined over time as well.

In another cross-sectional survey [1] there was a tendency for QoL issues to increase from the MHNW to the MHO, metabolically unhealthy normal weight, and MUHO groups among 6057 Korean population members. There is an increase in the number of people with inadequate sleep times from the MHNW to the MUHO (*P* for trend = 0.015). MHO participants were more likely to experience stress than the other individuals (*P* = 0.013). Following adjustment for gender, age, smoking state, physical activity, alcohol consumption, household income, and history of comorbidities, the ORs for movability difficulties in the MHO and MUHO groups were 1.43 (95% CI 1.01–2.04) and 1.94 (95% CI 1.52–2.47), respectively. The adjusted ORs for difficulties with self-care and common activities in the MUHO group were 2.07 (95% CI 1.39–3.10) and 2.08 (95% CI 1.53–2.81), respectively. The adjusted ORs for pain and displeasure in the MHO and MUHO groups were 1.35 (95% CI 1.06–1.73) and 1.39 (95% CI 1.14–1.69), respectively. Regarding psychiatric symptoms, after adjustment for the probable confounders, the adjusted OR for insufficient sleep duration in the MUHO group was 1.25 (95% CI 1.04–1.50) and the adjusted ORs for stress in the MHO and MUHO groups were 1.27 (95% CI 1.05–1.54) and 1.33 (95% CI 1.11–1.60), respectively. After controlling for the confounders, the mean EQ-5D scale in the MHO and MUHO groups were significantly lower than that of the metabolically healthy normal weight group (1.032–0.101 and 1.023–0.101 vs. 1.042–0.097, *P* = 0.011 and < 0.001, respectively). However, metabolically unhealthy normal weight and MHNW groups did not differ in mean EQ-5D scores.

The study by Tasi et al. [6] found that those with overweight/obesity and MetS had significantly lower scores on two subscales of the SF-36 (short form-36). These subscales evaluated aspects of mental well-being or scored the mental component summary. These were general health (*P* = 0.007) and physical functioning (*P* = 0.021). No difference was found between individuals with and without MetS on any of the four subscales of the SF-36 that evaluates mental health aspects or the mental component summary score.

A cross-sectional data [22], in 6860 men and women, from the German Health Interview and Examination Survey 2008–11, it was shown that compared to MHNW, all obese subgroups with different metabolic health had considerably lower physical component summary (PCS) score in men and women. a reverse relationship with PCS was strongest for MUHO (men: − 7.0 [− 8.2; − 5.8]; women: − 9.0 [− 10.2; − 7.9]), intermediate for metabolically unhealthy non-obese (men: − 4.2 [− 5.3; − 3.1]; women: − 5.6 [− 6.8; − 4.4]) and least pronounced for MHO (men: − 2.2 [− 3.6; − 0.8]; women − 3.9 [− 5.4; − 2.5]). Following adjustment for covariates, the MHNW variation is statistically significant for all groups, but declines for metabolically unhealthy non-obese (men: − 1.3 [− 2.3; − 0.3]; women: − 1.5 [− 2.7; − 0.3].

According to another work [12] conducted over 30 years which involved 6217 men and 8243 women, those with metabolically unhealthy normal weights were consistently sicker on all aspects and had poorer HRQoL than normal weight men. But, no significant influence observed after adjustment for possible confounders. Most adversely affected were the MUHO women, followed by the MHO women. In the MUHO and MHO groups, the variables related to mobility and disturbed HRQoL were significant after adjustment for all confounders.

In a research, conducted by UI-Haq et al. [31], revealed that as BMI increased, utility scores decreased in overweight/obese subjects with metabolic abnormalities (morbidly obese, adjusted coefficient: − 0.064, 95% CI − 0.115, − 0.012, *P* = 0.015 for metabolic comorbidity vs. − 0.042, 95% CI − 0.067, − 0.018, *P* = 0.001 for those without metabolic abnormality).

In another research [29] it was not observed any significant difference in HR-QoL between MHO and MUHO (SF-36 total score: 60 ± 20.8 vs. 62.8 ± 18.2, *P* = 0.27).

Lopez-Garcia et al. [5] stated that in comparison to MHNW subjects, the unhealthy normal-weight and the healthy overweight subjects had a similar PCS score; however, the PCS was lower among those with unhealthy overweight (− 1.79; 95% CI − 2.66 to − 0.94), with MHO (− 1.45; 95% CI − 2.67 to − 0.24) and unhealthy obesity (− 1.97; 95% CI − 2.88 to − 1.05). Regardless of metabolic condition, overweight or obesity did not affect the Mental Component Summary score.

In another study [32] between 2880 healthy adults with age > 19 years, Amiri et al. found that only physical aspects of HRQoL differ between obesity phenotypes, both in men and in women (*P* < 0.05). Additionally, following adjustment for marital state, age, job status, physical activity, and education, the likelihood of reporting poor physical HRQoL was considerably greater in both men (OR 1.960, 95% CI 1.037 ± 3.704; *P* < 0.05) and women (OR 2.887, 95% CI 1.674 ± 4.977; *P* < 0.001) with MUHO state, in comparison to MHNW individuals. However, with the exception of overweight women with normal metabolic state, who were less probably to have poor psychological wellbeing (OR 0.638, 95% CI 0.415 ± 0.981; < 0.05), mental HRQoL was not associated with either phenotype regardless of gender.

## Discussion

The current systematic review aimed to investigate mental health status and HRQoL in different obesity phenotypes. We found that when obesity coexists with metabolic disorders, its connection with mental health issues and poor QoL is more pronounced. However, in terms of mental disorders and poor quality of life, MHO is not fully benign.

As well as being associated with a variety of chronic diseases and metabolic disorders, obesity is also associated with one's mental health and quality of life [1]. There are some bidirectional relationships between MetS and depressive symptoms [33], and diabetes and depressive mood [34], proposing that a number of pathways may be involved in the association between excessive weight, metabolic disturbances, and depression.

There have been similar findings in studies of Canadian women [35] and Mexican men [36], indicating that excess weight does not predict depression. Further, MetS has not been associated with depression in Turkish adults [37]. In a follow-up study of metabolic phenotype in depression with long-term duration, depression risk was initially higher in metabolically unhealthy individuals regardless of weight status; however, this finding was not significant in MHO groups [30]. Among adults with obesity, metabolically healthy individuals have lower rates of depression and anxiety, whereas metabolically unhealthy patients have higher rates of depression and anxiety [7]. Although a previous meta-analysis concluded that obesity is associated with an elevated depression risk [9], the evidence is contradictory [10, 11, 38]. In relation to depression in obesity phenotypes, numerous factors, including metabolic factors, need to be investigated. After controlling for covariates such as gender, age, marital status, and education, these results remained the same. Previously, the relationship between obesity phenotypes and depressive mood was shown to be mediated by waist circumference (WC) and fasting blood sugar [28, 39]. Understanding the relationship between depressive mood and obesity phenotypes will require a deeper description of other covariates such as adipocytokine.

Studies suggest that certain physiological mechanisms may explain the elevated depression risk in MUHO people. Hypothalamic–pituitary–adrenal (HPA) axis disruptions may lead to dysfunction of cortisol regulation, culminating in dysglycemia and insulin resistance, causing a cascade of events in the MetS [27]. Depression is linked to disruptions of the HPA axis [27]. Additionally, depressive mood may occur in adolescents with obesity due to biochemical changes caused by metabolic disturbances, including expanded cerebrospinal fluid space and diminished white matter volume [27]. Furthermore, neurodegeneration and structural remodeling may impair emotion, study and memory through brain inflammation [40], and mostly affected the hippocampus [40]. A major component of the MUHO definition consisted of inflammation in the majority of previous studies. Accordingly, inflammation is a likely factor that contributes to the association between MUHO and depression, and the role of inflammatory agents and metabolic disturbances will continue to be determined [23].

MetS and work-related stress were linked in a previous systematic review [41]. In a meta-analysis [33], it was concluded that MetS is a risk factor for depression, and depression is a risk factor for MetS, demonstrating a bidirectional relationship. Metabolic disturbances are a major component of MetS, and these disturbances may be causing depression through their interactions with MetS. When it comes to metabolic abnormalities and depression, age plays a major role in moderating the relationship. Various combinations of MetS components have different effects on mortality risk based on the age at which MetS presents and when the MetS component presents [42]. It is probable that metabolic dysfunction may also be linked to depression based on age differences in its manifestation. Further, the theory holds that a higher BMI may be indicative of greater physiologic and functional reserve (due to greater muscle mass), preventing depression later in life [43].

The main marker for MetS, insulin resistance derived from excess adiposity and persistent low-grade inflammation, is more widely developed as a result of hypertension, dyslipidemia, and an inflammatory state [7]. Depressive disorders have been related to some MetS components, as well with disruptions of metabolic networks, like insulin-glucose homeostasis, inflammatory processes, and unhealthy lifestyle behaviors. [7, 9, 44–46]. According to an epidemiological study, depression is twofold more common among diabetic patients than in the general population, and diabetes increases the risk of depression by twofold [47]. This relationship has also been reported to be explained by the inefficient utilization of glucose caused by central insulin resistance in vulnerable brain regions (such as limbic system) in depressed patients [24]. In animal models, it has been shown that brain-specific knockout of insulin receptor in mice elevates age-associated anxiety and depressive-like behavior by altering dopamine metabolism [48]. The relationship was positive among younger subjects, but weakened as subjects aged [24]. It remains unclear what the logical basis is for this pattern, but it can be attributed to selection bias. A logical justification for this pattern is not clear, but it is identified that selection bias may be present. Most likely, older subjects with greater levels of depression and comorbidities refused to participate in the study.

Serum lipids and depression have been investigated, but so far the evidence has been inconsistent, and most studies have focused primarily on total cholesterol levels [49]. Depression was associated with a lower HDL cholesterol level [50]. Patients with bipolar depression had significantly higher levels of triglycerides (TG) than healthy controls [51]. The OR for low HDL cholesterol as well as hypertriglyceridemia in men suffering from severe depression was significantly higher, and the OR in women with hypertriglyceridemia was also significantly higher [52]. In addition, in contrast to people with remitted depression and healthy controls, those currently suffering from major depression have higher levels of TG and lower levels of HDL [53].

Depression and blood pressure have an inconsistent relationship. Low blood pressure and depression have been linked in some cross-sectional studies [54], while longitudinal studies concluded that depression predicted low blood pressure [55]. Low blood pressure, on the other hand, was a predictor of higher depression levels [56]. Several studies found that late-life depression and high blood pressure were related [57]. Hence, it would be good to study the Vascular Depression hypothesis [58], according to which certain geriatric depressive disorders are predisposed to, accelerate, or continue as a result of cardiovascular disease, such as high blood pressure.

The sex-related differences can be justified by leptin concentration. Based on the gender of the subjects, the concentration of serum leptin and depression had different relations. Women with depression had elevated leptin levels, but men did not [59]. In another study, serum levels of leptin were found to be high in all people with depressive symptoms, but higher in women who had depression than men with depressive symptoms [60].

In light of the diverse range of physical and psychosocial factors affecting obesity subtypes and depression, differences between the findings may have their roots in several factors. Other than gene-environment interactions [61], psychological factors also appear to have an impact on this relationship. Furthermore, this inconsistency may result from the diversity in the definition of MUHO due to the lack of universal agreement. A previous study involving three different definitions for metabolic health found that the association between excess weight and mood varied widely according to the definition of metabolic health [7]. Furthermore, the study designs or follow-up durations differed. Moreover, it is impossible to prove reverse causality or residual confounding from observational evidence even when obtained from a longitudinal study. So, there might be a relationship between abnormal metabolic condition and depression due to unmeasured risk behaviors such as poor diet or noncompliance with medical treatment [30].

Aside from mental challenges, obesity is also associated with disability and comorbidity problems, which can adversely affect one's QoL. Only the physical health domain of the SF-36 index was associated with MetS and poor QoL in a cross-sectional study; as soon as BMI was controlled for, the relationship disappeared, indicating that BMI alone accounts for the relationship [6] proposing that the extent of metabolic abnormalities may not be linearly associated with such mental issues and QoL. Excess weight led to the greatest impairment in mobility in both men and women [62]. A lower HRQoL was related to the physical functioning feature in obese males and females [63, 64]. There is inconsistent evidence that metabolic health affects Euroqol-5 dimensions (EQ-5D); pain/discomfort showed a higher OR in those with MetS [65]. The male population with MetS had disturbances in usual activities, but the female population with MetS showed difficulties in all 5 aspects of EQ-5D [14]. Fatigue and excessive daytime sleepiness are also main symptoms associated with central obesity, and may negatively impact women's HRQoL [66]. There may be some explanation for the lower HRQoL of women than men based on sex-different prevalence of obesity-induced comorbidities and health-related behaviors such as physical activity [22]. However, as revealed by a cross-sectional study [12], in which HRQoL were assessed using the EQ-5D questionnaire, physical aspect of HRQoL were lower among metabolically unhealthy non-obese participants, suggesting that metabolic issues are more influential than excessive weight.

According to our knowledge, this is the first study to look systematically at the relationship between obesity phenotypes and mental health and HRQoL. However, this review is limited by the small number of researches. There are a multitude of outcome measurements reported across age groups and with a variety of genetic variations contributing to heterogeneity. The relation was not evaluated for secular direction since few studies examined the relationship longitudinally. It is therefore necessary to conduct large longitudinal cohort studies to clarify this association.

## Conclusion

Overall, it is reasonable to conclude that when obesity occurs in conjunction with metabolic disturbances, its relationship with mental health issues and poor QoL is strengthened. In order to decrease the heavy burden of comorbid depression in obese individuals, we need to better understand the relationship between obesity phenotypes and mental health and HRQoL. These strategies may include pharmacological (such as anti-inflammatory medications and/or surgically-induced weight loss) or non-pharmacological interventions (such as weight loss programs and nutritional interventions with immunomodulatory effects) aimed at decreasing metabolic abnormalities and systemic inflammation in obese patients.

## Data Availability

Data sharing is not applicable to this article as no datasets were generated or analysed during the current study.
